# Supplementary material for: Development of Machine Learning Models Predicting Estimated Blood Loss during Liver Transplant Surgery
Source: J Pers Med. 2022 Jun 23;12(7):1028. doi: 10.3390/jpm12071028 (PMC9320884; doi:10.3390/jpm12071028)
Supplement: Supplementary file 1 [file jpm-12-01028-s001.zip › supplementary tables.pdf]

Supplementary Table S1. Included 59 features

Ranking	Features
1	Hepatocellular carcinoma
2	Activated partial thromboplastin time (aPTT)
3	Blood urea nitrogen
4	Operation time
5	Body temperature
6	MELD (Model for End-Stage Liver Disease) Score
7	Mean arterial pressure
8	Creatinine
9	Pulse pressure
10	Hemoglobin
11	Trans-Arterial Chemo-Embolization (TACE)
12	Respiratory rate
13	Preoperative continuous renal replacement therapy
14	Fresh frozen plasma transfusion in the preoperative period
15	Albumin
16	INR(Prothrombin time)
17	Estimated Glomerular Filtration Rate
18	Hepatitis B virus carrier
19	Platelet count
20	Liver cirrhosis
21	Platelet concentration transfusion in the preoperative period
22	Alcoholic liver cirrhosis
23	Norepinephrine infusion rate (mcg/kg/min)
24	Surgical past history

25	SpO2
26	Anesthetic agent
27	Body mass index
28	Weight
29	Heart rate
30	IgG antibody titer
31	Huge collateral on CT
32	External beam radiation therapy
33	ABO incompatibility
34	Total intravenous anesthesia
35	Desflurane
36	Hepatitis C virus carrier
37	Heparin user
38	Portal vein thrombosis on CT
39	Age
40	Sex
41	Robot donor
42	Height
43	Radiofrequency Ablation
44	RBC transfusion
45	IgM antibody titer
46	Planned splenectomy
47	Emergency
48	Living or deceased donor
49	Taking only aspirin
50	Taking both aspirin and clopidogrel
51	Taking only clopidogrel
52	Taking lixiana

53	Taking cilostazol
54	Anesthesia with both sevoflurane and desflurane
55	Vasopressin infusion rate (unit/hr)
56	Yttrium-90 Selective Internal Radiation Therapy
57	Intra-arterial Chemotherapy
58	Narrow portal vein on CT
59	Hepatic artery thrombosis on CT

Supplementary Table S2. machine learning model specifics.

Method	Hyperparameter	
	Model specification and search grids	Selected value
Elastic net	λ: 500 equally spaced values in logarithmic scale between 10^{-6} and 10^{10}<br>α: 0, 0.1, 0.2, 0.3, 0.4, 0.5, 0.6, 0.7, 0.8, 0.9, 1	λ: 0.1813498<br>α: 0
Random forests	mtry: 1, 2, 3, 4, 5, 6, 7, 8	mtry: 1
Extreme gradient boosting	Number of rounds: 1000 (early stopping with patience 20)<br>Maximum depth: 3, 5<br>Collection sample bytree: 0.7<br>eta: 0.05, 0.1<br>gamma: 0, 1, 2<br>Minimum sum of instance weight (hessian) needed in a child : 3, 5<br>subsample: 0.5	Iteration : 21<br>Maximum depth: 3<br>Collection sample bytree: 0.7<br>eta: 0.05<br>gamma: 2<br>Minimum sum of instance weight (hessian) needed in a child : 5<br>subsample: 0.5
Neural networks	Input layer with 8 nodes, two hidden layers with varying numbers of nodes with the relu activation, output layer of one node with the sigmoid activation, dropout with varying	Dropout: 0.3<br>Batch size: 32<br>Number of nodes in hidden

	rates applied for both hidden layers, and epochs set to 1000 with early stopping patience 20.<br><br>Dropout: 0.2, 0.3<br>Batch size: 8, 16, 32,<br>Number of nodes in hidden layers: 10, 15, 20.	layers: 10
