# Supplementary figures and images for: Development of Machine Learning Models Predicting Estimated Blood Loss during Liver Transplant Surgery
Source: J Pers Med. 2022 Jun 23;12(7):1028. doi: 10.3390/jpm12071028 (PMC9320884; doi:10.3390/jpm12071028)

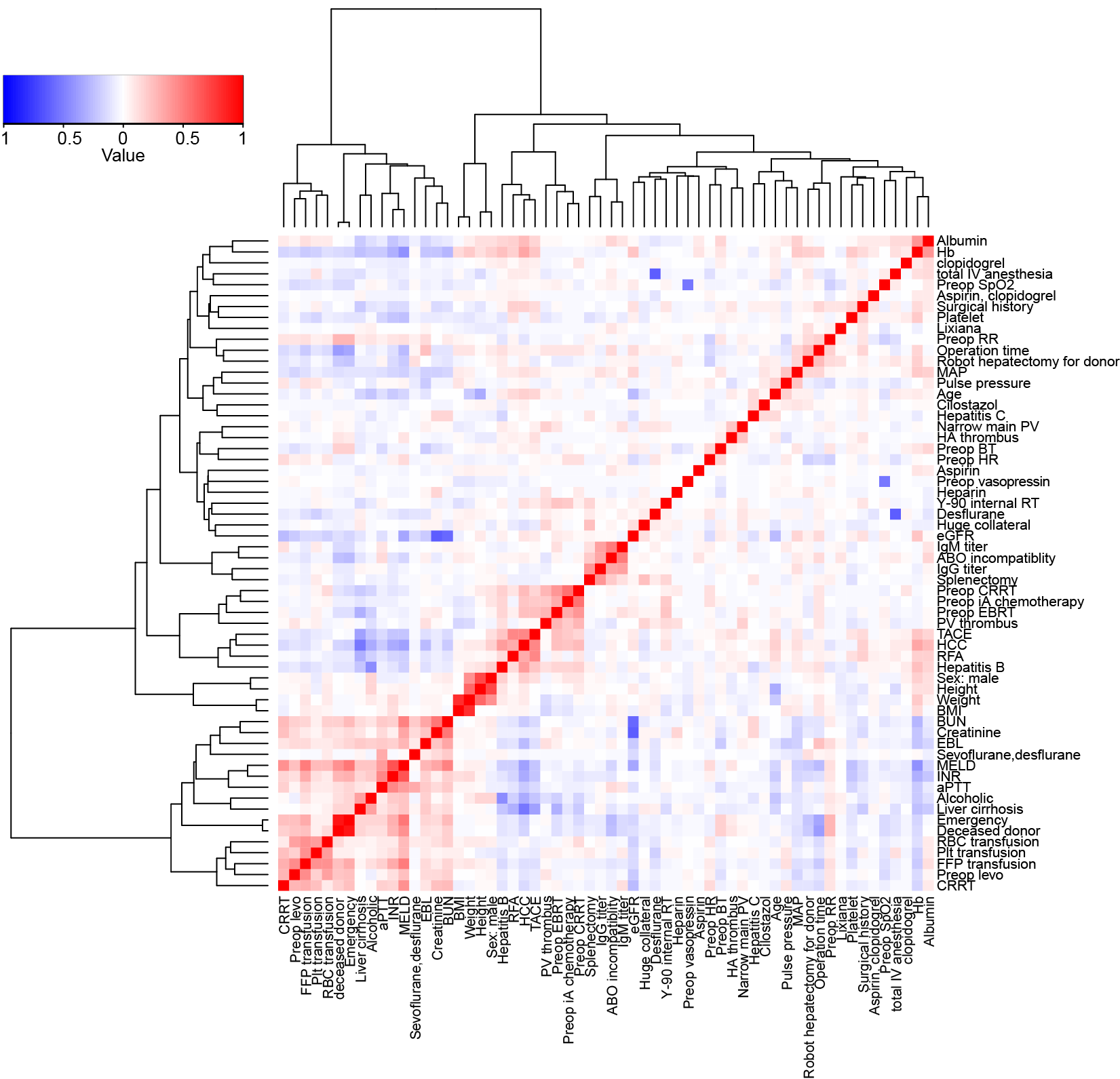

Supplement: Supplementary file 1 [file jpm-12-01028-s001.zip › Figure S1.tif]

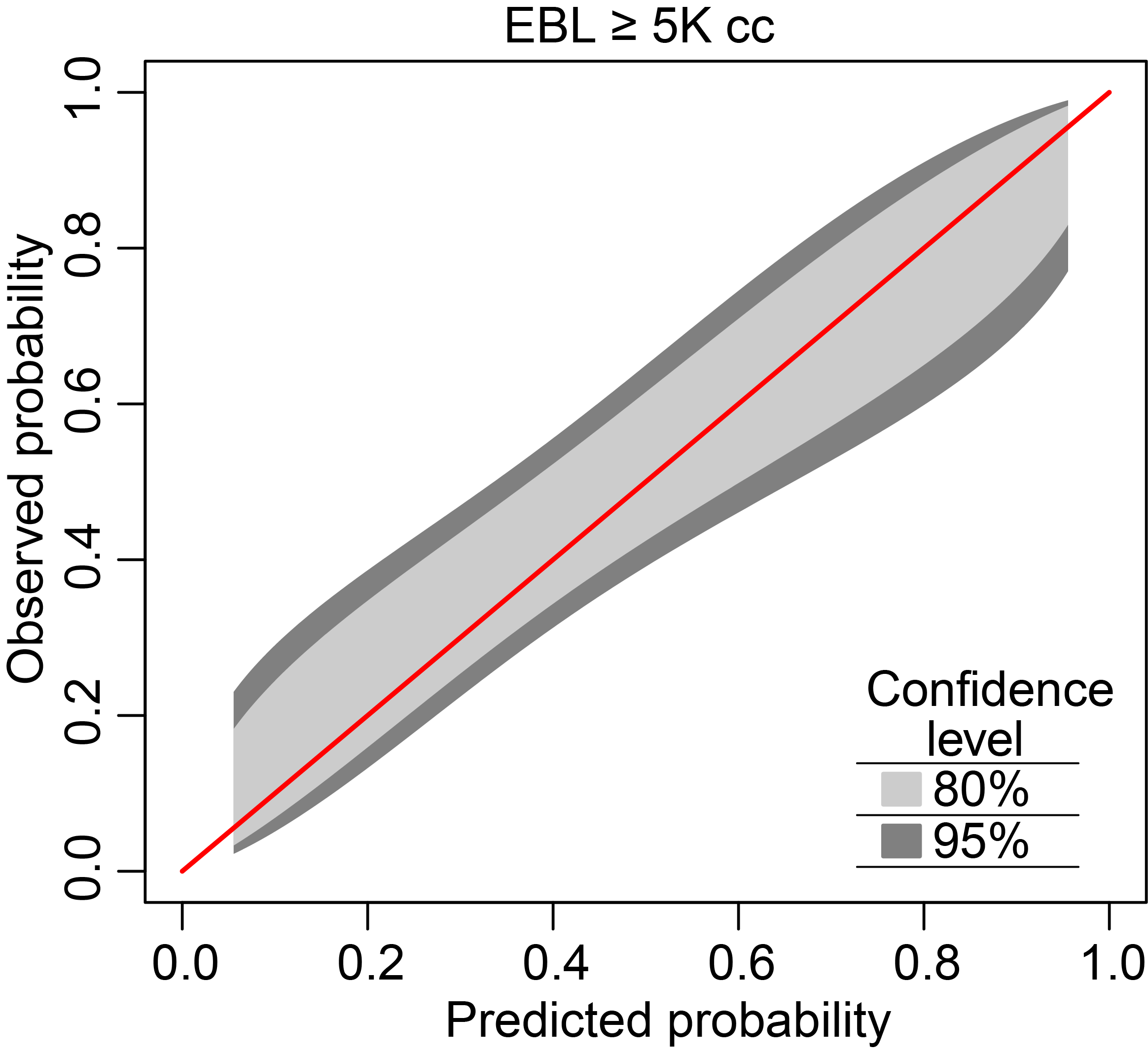

Supplement: Supplementary file 1 [file jpm-12-01028-s001.zip › Figure S2.tif]
